# Plant Aquaporins in Infection by and Immunity Against Pathogens – A Critical Review

**DOI:** 10.3389/fpls.2019.00632

**Published:** 2019-05-28

**Authors:** Liyuan Zhang, Lei Chen, Hansong Dong

**Affiliations:** ^1^Plant Immunity Research Group, National Key Laboratory of Crop Science, Department of Plant Pathology, Shandong Agricultural University, Tai’an, China; ^2^Plant Immunity Laboratory, Department of Plant Pathology, Nanjing Agricultural University, Nanjing, China

**Keywords:** aquaporin, plasma membrane intrinsic protein, H_2_O_2_ transport, immunity signaling, translocon, type III effectors

## Abstract

Plant aquaporins (AQPs) of the plasma membrane intrinsic protein (PIP) family face constant risk of hijack by pathogens aiming to infect plants. PIPs can also be involved in plant immunity against infection. This review will utilize two case studies to discuss biochemical and structural mechanisms that govern the functions of PIPs in the regulation of plant infection and immunity. The first example concerns the interaction between rice *Oryza sativa* and the bacterial blight pathogen *Xanthomonas oryzae* pv. *oryzae* (Xoo). To infect rice, Xoo uses the type III (T3) secretion system to secrete the proteic translocator Hpa1, and Hpa1 subsequently mediates the translocation of T3 effectors secreted by this system. Once shifted from bacteria into rice cells, effectors exert virulent or avirulent effects depending on the susceptibility of the rice varieties. The translocator function of Hpa1 requires cooperation with OsPIP1;3, the rice interactor of Hpa1. This role of OsPIP1;3 is related to regulatory models of effector translocation. The regulatory models have been proposed as, translocon-dependent delivery, translocon-independent pore formation, and effector endocytosis with membrane protein/lipid trafficking. The second case study includes the interaction of Hpa1 with the H_2_O_2_ transport channel AtPIP1;4, and the associated consequence for H_2_O_2_ signal transduction of immunity pathways in *Arabidopsis thaliana*, a non-host of Xoo. H_2_O_2_ is generated in the apoplast upon induction by a pathogen or microbial pattern. H_2_O_2_ from this source translocates quickly into Arabidopsis cells, where it interacts with pathways of intracellular immunity to confer plant resistance against diseases. To expedite H_2_O_2_ transport, AtPIP1;4 must adopt a specific conformation in a number of ways, including channel width extension through amino acid interactions and selectivity for H_2_O_2_ through amino acid protonation and tautomeric reactions. Both topics will reference relevant studies, conducted on other organisms and AQPs, to highlight possible mechanisms of T3 effector translocation currently under debate, and highlight the structural basis of AtPIP1;4 in H_2_O_2_ transport facilitated by gating and trafficking regulation.

## Introduction

Aquaporins (AQPs) are membrane-intrinsic proteins initially defined as water (H_2_O) transporting channels in all organisms and subsequently found to have many other substrate specificities (de Groot and Grubmuller, 2001; Maurel et al., 2008, 2015; Sutka et al., 2017), such as hydrogen peroxide (H${}_{2}$O${}_{2}$; Tian et al., 2016). In plants, AQPs are classified into five major families (Chaumont et al., 2001; Maurel, 2007), including the plasma membrane intrinsic proteins (PIPs), tonoplast intrinsic proteins (TIPs), nodulin 26 like intrinsic proteins (NIPs), small basic intrinsic proteins (SIPs), and X intrinsic proteins (XIPs). The PIP family is further divided into the PIP1 subfamily made of PIP1;1 to PIP1;5 and the PIP2 subfamily consisting of PIP2;1 to PIP2;8 in most plant species (Maurel, 2007; Gomes et al., 2009; Laloux et al., 2018). While AQPs of the other four families function in substrate trafficking between organelles, PIPs are responsible for substrate transportation between the exterior and interior of cells (Maurel, 2007; Gomes et al., 2009; Kaldenhoff et al., 2014; Li et al., 2015; Bao, 2017).

Recently discovered functions of AQPs surpass the original “water channel” concept (Preston et al., 1992; Wudick et al., 2009; Heckwolf et al., 2011; Brown, 2017), and suggest implications in infection and immunity in both animals (Hara-Chikuma et al., 2015; Yang, 2017) and plants (Maurel et al., 2015; Wang X. et al., 2018; Zhang et al., 2018; Li et al., 2019). The functions of animal AQPs are no longer confined to substrate-transport-based processes such as urinary concentration and body fluid homeostasis (Brown, 2017), and are now known to include roles in various disease conditions and pathological states (Yang, 2017). Similarly, functional diversity – redundancy, overlapping, and extension beyond substrate transport – is a property of plant AQPs, especially PIPs (Ji and Dong, 2015b; Li et al., 2015; Zhang et al., 2018, 2019; Li et al., 2019). The functional scope of PIPs goes far beyond water relations or drought tolerance, extending to the subcellular transport of reactive oxygen species (ROS), including H_2_O_2_ (Tian et al., 2016; Smirnoff and Arnaud, 2019). H_2_O_2_ transport connects with signaling between the cell exterior and interior and between organelles, resulting in plant resistance to pathogen infection (Tian et al., 2016).

PIPs possess extracellular regions exposed to the outside environment (Maurel et al., 2015), and have potential to partake in plant responses to biotic and abiotic stresses. Here are several examples. Previous uses of induced resistance in crop protection (for example: Chen et al., 2008a, b; Fu et al., 2014; Wang F. et al., 2014) confirm the practical value of PIP-mediated immunity signal transduction (Tian et al., 2016). The correlation of PIP function in water transport with stress response results in promising strategies for improvement of plant tolerance to abiotic stresses, including drought (Balestrini et al., 2018). Drought tolerance in a variety of plant species is related to arbuscular mycorrhizal (AM) symbiosis, in which AM fungi (*Rhizophagus* spp.) show enhanced expression of AQP-encoding genes (Bárzana et al., 2014, 2015; Calvo-Polanco et al., 2016; Ruiz-Lozano et al., 2016; Sánchez-Romera et al., 2016; Ruiz-Lozano and Aroca, 2017). Surprisingly, the AM fungus *R. clarus* contributes its aquaglyceroporin (glycerol/water-transporting AQP) RcAQP3 to the mediation of long-distant polyphosphate translocation from the fungal vacuoles into cells of plant roots and leaves (Kikuchi et al., 2016). Genetic resources of plants, including the AQP transcriptome, can be used in responses to environmental cues, symbiotic microbes (AM fungi and rhizobia), and microbial pathogens (Desaki et al., 2018; Rey and Jacquet, 2018; Wang R. et al., 2018).

Due to their direct contact with the extracellular environment, PIPs risk being appropriated by plant pathogens to expedite infection (Zhang et al., 2018; Li et al., 2019). When infection is imminent, the real-time function of PIPs may switch from substrate transport to the regulation of plant responses to pathogens (Zhang et al., 2018; Li et al., 2019). This is either favorable or unfavorable to plant growth and development, depending on plant responses to pathogenicity determinants, called effectors, whose functions are subject to regulation of PIPs (Tian et al., 2016; Wang X. et al., 2018; Li et al., 2019; Zhang et al., 2019).

This review will summarize recent studies on the roles of PIPs in plant infection and immunity, and discuss the molecular, biochemical, and structural mechanisms involved. Discussion of infection will focus on type III (T3) effector translocation (T3ET) from *Xanthomonas oryzae* pv. *oryzae* (Xoo) into rice cells. Discussion of immunity will focus on the response of Arabidopsis to pathogens or pathogen-associated molecular patterns (PAMPs), also termed microbial patterns. This review will reference studies investigating AQPs in animals, microbes, and other plants to highlight the broad importance of PIP function, from substrate transport to infection and immunity in plants.

## The Circumstantial Function of a PIP in T3ET

PIPs possess three extracellular regions that are exposed to the outside environment (Maurel et al., 2015). As a result, they are at a constant risk of being hijacked by pathogens attempting to infect plants, and inevitably partake in immunity against infection. Therefore, PIPs are required to extend their function from substrate transport to plant infection and immunity when the circumstances demand it. Emerging evidence suggests the implication of OsPIP1;3 in rice infection by Xoo (Zhang et al., 2018; Bian et al., 2019; Li et al., 2019). In this case, OsPIP1;3 functions with the bacterial hydrophilic protein Hpa1, which belongs to the harpin-group proteins secreted by the T3 secretion pathway of Gram-negative plant-pathogenic bacteria (Wei et al., 1992; Tejeda-Dominguez et al., 2017; Zhang et al., 2018; Li et al., 2019). Hpa1 produced by *X. oryzae* (Zhu et al., 2000; Chen et al., 2008a) is involved in the virulence of bacterial pathogens (Wang X. et al., 2018). Hpa1 modulates physiological and pathological processes in plants in association with PIPs (Sang et al., 2012; Li et al., 2013, 2014, 2015, 2019; Ji and Dong, 2015b; Zhang et al., 2018). The virulence role of Hpa1 is determined by its biochemical properties. Hpa1 is a one-domain harpin, which share a unitary hydrophilic “harpin” domain distinct from the enzymatic domain present in two-domain harpins (Kvitko et al., 2007; Choi et al., 2013; Ji and Dong, 2015b). Two-domain harpins have potential to associate with the bacterial periplasm or plant cell walls to facilitate assembly of the T3 secretion machinery (Mushegian et al., 1996; Koraimann, 2003; Zhang et al., 2008; Dik et al., 2017; Hausner et al., 2017). One-domain harpins, including Hpa1, target plasma membranes (PMs), where they serve as T3 translocators to mediate T3ET (Kvitko et al., 2007; Bocsanczy et al., 2008; Wang X. et al., 2018; Bian et al., 2019; Li et al., 2019).

In Xoo-infected rice plants, secreted Hpa1 translocates at least two transcription activator-like (TAL) effectors – AvrXa10 and PthXo1, which are also produced via the pathway (Wang X. et al., 2018). Translocated effectors exert virulent or avirulent effects depending on the susceptibility of the plant variety (Yang et al., 2006; Büttner, 2016; Schreiber et al., 2016; Schwartz et al., 2017; Zhang et al., 2018). The rice variety Nipponbare is susceptible to the TAL effector PthXo1 secreted by PXO99^A^, a well-studied Xoo strain (Yang et al., 2006; Wang X. et al., 2018; Zhang et al., 2018). To infect Nipponbare plants, PXO99^A^ secretes Hpa1 and delivers it to the cell surface, where Hpa1 interacts with OsPIP1;3 to facilitate the translocation of subsequently secreted PthXo1 into Nipponbare cells (Wang X. et al., 2018; Li et al., 2019). PthXo1 then induces virulence by activating its regulatory target – the host susceptibility gene *OsSWEET11* (Yang et al., 2006) in an OsPIP1;3-dependent manner (Zhang et al., 2018; Li et al., 2019). If the *OsPIP1;3* gene is silenced by hairpin or knocked out by TALEN^14^, both PthXo1 translocation and *OsSWEET11* expression incur concomitant impairments up to 70%, highly alleviating virulence as a consequence (Zhang et al., 2018; Li et al., 2019). In contrast, both events acquire >2-fold enhancements if *OsPIP1;3* is overexpressed, causing marked aggravations in virulence (Li et al., 2019).

AvrXa10 is an avirulent effector secreted by the Xoo strain PXO86, and induces immune responses in the resistant rice variety IRBB10 (Tian et al., 2014). The plant immunity is determined by the disease-resistant gene *Xa10*, which is the target of AvrXa10 (Tian et al., 2014). *Xa10* has two homologs in the Nipponbare genome – *Xa10-Ni* and *Xa23-Ni*, both of which function similarly to confer immune responses in Nipponbare plants inoculated with recombinant PXO99^A^ strains that deliver the matching artificially designed TAL effectors (Wang et al., 2017). When *avrXa10* is transferred from PXO86 into the PXO99^A^ genome, the resulting PXO99^A^/*avrXa10* recombinant delivers AvrXa10 in IRBB10 cells (Wang X. et al., 2018). Thereafter, AvrXa10 activates the disease resistant gene *Xa10-Ni* to confer the plant resistance against the blight disease (Wang X. et al., 2018). The AvrXa10 translocation and *Xa10-Ni* activation incur concomitant impairments in plants inoculated with the *hpa1*-deleted mutant; the absence of *hpa1* markedly reduces the quantity of AvrXa10 translocation, decreasing the expression level of *Xa10-Ni* (Wang X. et al., 2018). The AvrXa10 translocation and *Xa10-Ni* activation requires *OsPIP1;3*, and both events are enhanced by *OsPIP1;3* overexpression but inhibited by *OsPIP1;3* silencing (Bian et al., 2019).

These findings demonstrate the important role of OsPIP1;3 in the translocation of T3 effectors, at least the TAL effectors PthXo1 and AvrXa10, from bacterial cells into the cytosol of rice cells. OsPIP1;3 functions either as a disease-susceptibility or -resistance factor, depending on a virulent or avirulent function of the translocated effector.

## Potential Mechanisms of T3ET Regulation

**FIGURE 1:**
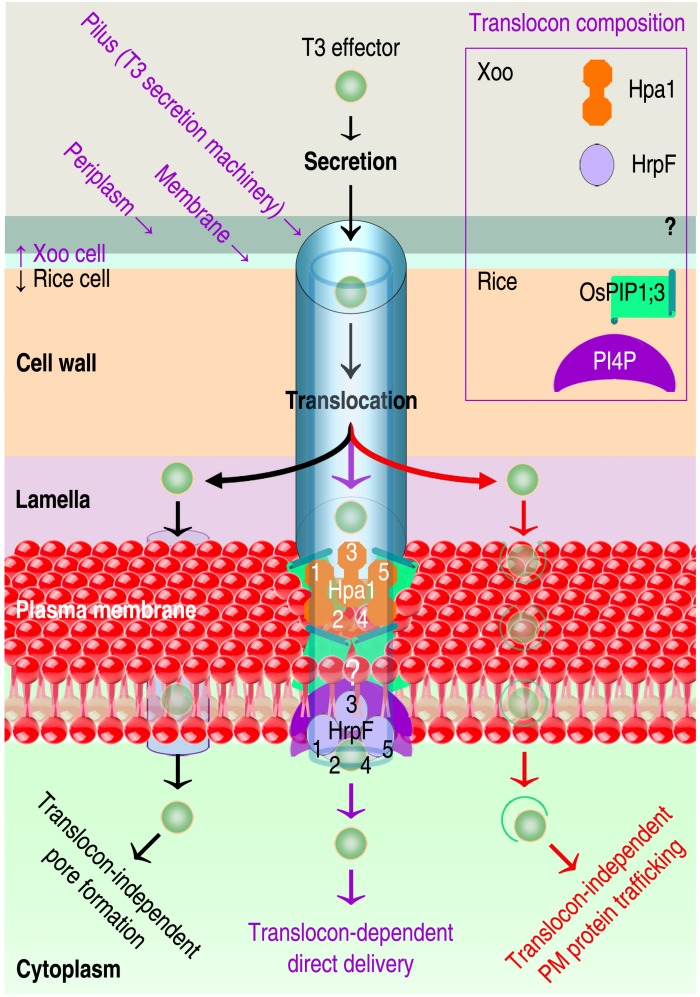
Hypothetic routes of T3ET using Xoo as an example. Effector translocation may use the left black route (Rüter et al., 2010; Scheibner et al., 2017) or the right red pathway (Lu et al., 2007; Santi-Rocca and Blanchard, 2017) according to recently proposed models. In a previously proposed model, T3ET occurs via the translocon (the middle purple route) hypothetically assembled by interactions between translocators, and their receptors in eukaryotic PMs (Büttner, 2012; Ji and Dong, 2015b). Three translocators have been identified in animal-pathogenic bacteria, but the number of T3 translocator remains unknown in plant-pathogenic bacteria including Xoo. In Xoo, the hydrophilic protein Hpa1 (Wang X. et al., 2018) and the hydrophobic protein HrpF (Büttner et al., 2002; Li et al., 2011) were determined to function as T3 translocators, but whether the third translocator exists is unclear (question marks). Regarding molecular interactions during the translocon assembly, OsPIP1;3 has been verified to interact with Hpa1 at rice PMs to expedite the translocation of TAL effectors AvrXa10 and PthXo1 (Zhang et al., 2018; Li et al., 2019). In the cartoon, numbers 1 through 5 refer to the order of the translocator in self oligomerization to form the homogenous complex, which is assumed to be consisting of 5 or 8 monomers (Mueller et al., 2008). *In vitro* assays indicated HrpF binding to lipids (Büttner et al., 2002; Li et al., 2011), such as PI4P, but no evidence was available to demonstrate the lipid binding at plant PMs and the subsequent effect on T3 effector translocation.

Passages of proteic T3 effectors are 1.2–5.0 nanometers in width (Ji and Dong, 2015b; Guignot and Tran Van Nhieu, 2016), in contrast to PIP/AQP channels with an aperture around 3 Å, which is permeable to small substrates (Heckwolf et al., 2011; Li et al., 2015; Tian et al., 2016) but impossible for proteins to pass (Li et al., 2019). Presumably, the role of OsPIP1;3 in T3ET complies with one of regulation models currently in debate (Domingues et al., 2016; Prasad et al., 2016; Santi-Rocca and Blanchard, 2017; Scheibner et al., 2017; Tejeda-Dominguez et al., 2017; Gaytán et al., 2018; Wagner et al., 2018; Shanmugam and Dalbey, 2019). Three models have been proposed as the canonical translocon-dependent delivery (Büttner, 2012; Figure 1 middle purple route) and the translocon-independent pore formation (Rüter et al., 2010; Figure 1 left black route) and endocytosis (Santi-Rocca and Blanchard, 2017; Figure 1 right red route). To date, studies on the three models have obtained empirical genetic evidence (Finsel and Hilbi, 2015; Domingues et al., 2016; Dong et al., 2016; Chakravarthy et al., 2017; Scheibner et al., 2017), but the structural basis of each model remains to be analyzed.

The first model of T3ET (Figure 1 middle purple route) was proposed to emphasize molecular interactions between T3 translocators and molecular interactions of T3 translocators with PM receptors (Büttner and Bonas, 2002; Büttner et al., 2008; Büttner, 2012; Ji and Dong, 2015b), either lipids (Haapalainen et al., 2011; Li et al., 2011), or proteins (Oh and Beer, 2007; Li et al., 2015; Adam et al., 2017). T3 translocators include one hydrophilic protein, such as Hpa1 from xanthomonads – bacteria in the *Xanthomonas* genus (Zhu et al., 2000; Chen et al., 2008a; Wang X. et al., 2018), and two hydrophobic proteins (Büttner et al., 2008; Ji and Dong, 2015b), such as HrpF from the same bacteria (Büttner et al., 2002; Li et al., 2011; Hausner et al., 2017). Recognition of the hydrophilic translocator by a component of the PM composition is the first step towards translocon assembly (Goure et al., 2004; Mueller et al., 2008; Sawa et al., 2014). Then, the translocon is finalized by the binding of lipids to hydrophobic translocators (Büttner et al., 2008; Büttner, 2012; Ji and Dong, 2015b). A completed translocon possesses an inner conduit that opens into a target cell and accommodates bacterial effector translocation (Büttner et al., 2008; Chatterjee et al., 2013; Ji and Dong, 2015b; Büttner, 2016).

Although there is no evidence so far to verify the T3 translocon assembly, many studies suggest the involvement of T3 translocators in effector translocation from animal- and plant-pathogenic bacteria into cells of their corresponding eukaryotic hosts (summarized in Scheibner et al., 2017). Mounting evidence indicates the engagement of PM phospholipids in T3ET, especially phosphatidylinositol phosphates PI(n)Pn (Lee et al., 2001a, b; Büttner et al., 2002; Weber et al., 2006; Hubber and Roy, 2010; Li et al., 2011; Finsel and Hilbi, 2015; Dong et al., 2016). For T3ET from xanthomonads, lipids in the plant PM associates with the bacterial hydrophobic T3 translocator HrpF (Büttner et al., 2002; Li et al., 2011). HrpF was the first reported T3 translocator and is regarded as a marker of T3 translocon in xanthomonads (Büttner et al., 2002; Scheibner et al., 2017). HrpF is highly conserved in xanthomonads (Sugio et al., 2005) and has been shown to mediate the translocation of AvrBs3 from *X. campestris* pv. *vesicatoria* (*Xcv*) – the bacterial spot pathogen of pepper (Büttner et al., 2002; Noël et al., 2002), and from *X. oryzae* pv. *oryzicola* — the pathogen that causes bacterial leaf streak in rice (Li et al., 2011). Evidence is further provided by our demonstrations that the hydrophilic T3 translocator Hpa1 of Xoo interacts with OsPIP1;3 at rice PMs to expedite translocation of TAL effectors PthXo1 and AvrXa10 from Xoo cells into the cytosol of rice cells (Zhang et al., 2018; Bian et al., 2019; Li et al., 2019).

The second model of T3ET (Figure 1 left black pathway) is the translocon-independent pore formation by bacterial effectors characteristic of cell-penetrating peptide (RPP; Scharnert et al., 2013; Rüter and Schmidt, 2017). Pore forming in eukaryotic PMs is momentary, occurs quickly upon recognition of bacterial effectors, and is regulated by membrane repair mechanisms (Scharnert et al., 2013). RPPs are either autonomously transported across the membrane or delivered by endocytosis (Wang F. et al., 2014). Autonomous translocation was found with the T3 effector YopM from *Yersinia enterocolitica* (Rüter et al., 2010). The YopM sequence contains two N-terminal α-helices, which determines the interaction with eukaryotic PMs (Li et al., 2019), and two putative nuclear localization signals at the C-terminus (Benabdillah et al., 2004). Therefore, YopM can be translocated directly into the cytosol of target cells and further transported into the nucleus via vesicle trafficking (Skrzypek et al., 1998).

Little is known about the translocon-independent translocation of T3 effectors from plant-pathogenic bacteria except for the TAL effector AvrBs3 from *Xcv*. Preliminary infection experiments with *Xcv* translocon mutants and endocytosis inhibitors deny a contribution of endocytosis to the delivery of AvrBs3 (Scheibner et al., 2017). A possible route for AvrBs3 translocation from the translocon mutants is a direct transportation through pore formation. The pore could be proteolipidic (Gilbert et al., 2014) and could be generated by means of proteic and lipidic constituents, which are required for the translocation of T3 effectors from xanthomonads (Büttner et al., 2002; Li et al., 2011, 2019). However, the efficiency of AvrBs3 translocation from the translocon mutants is much lower than that from the WT strain, indicating that the translocon-independent route is used in the absence of alternative.

**FIGURE 2:**
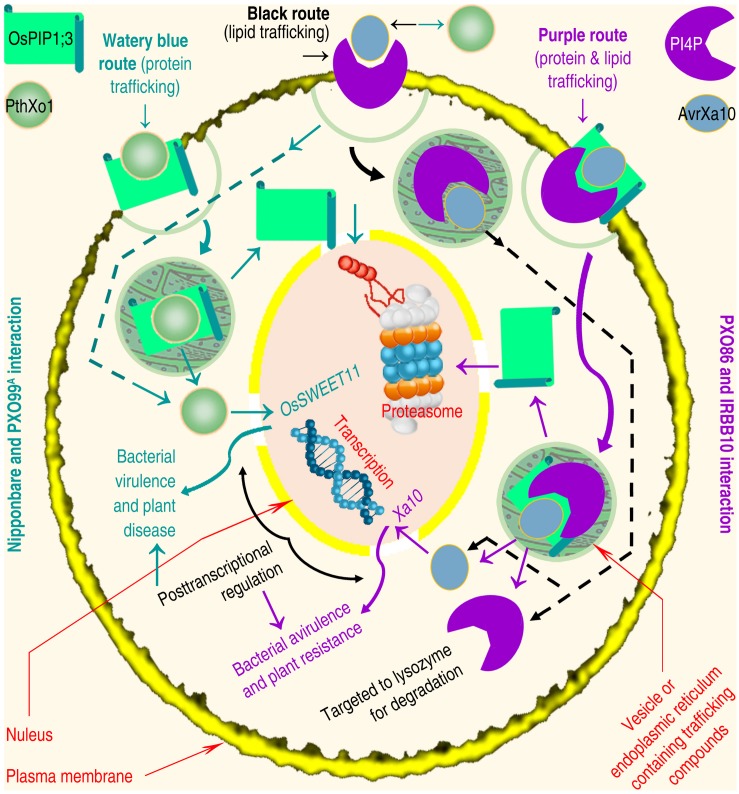
Diagram of hypothesized PM protein and lipid trafficking that is going through ER (Obacz et al., 2017) or vesicles (Wudick et al., 2015) and drives T3 effector endocytosis (Domingues et al., 2016). The OsPIP1;3-dependent and/or PI4P-involved PthXo1 and AvrXa10 translocation is used as a study paradigm. The protein and lipid trafficking pathways are annotated as a motivation for both effectors to be internalized and then both effectors execute the transcriptional regulation on their target genes. Both pathways may involve unannotated response, that is the recognition of Hpa1 by OsPIP1;3 and HrpF by PI4P (Büttner et al., 2002, Büttner, 2012; Ji and Dong, 2015b). The protein and lipid trafficking may be concurrent, cooperative or independent, making responses on PMs more intricate than the regular remodeling in the absence of bacterial proteins (Hubber and Roy, 2010; Piscatelli et al., 2016; Santi-Rocca and Blanchard, 2017).

The third model of T3ET (Figure 1 left black pathway) was recently proposed to emphasize the effector endocytosis through direct interaction with receptors situated in eukaryotic PMs (Domingues et al., 2016). The molecular interaction may trigger the membrane trafficking mechanism (Allgood and Neunuebel, 2018) either by endoplasmic reticulum (ER) or vesicles (Wudick et al., 2015), providing a potential scheme for bacterial effector endocytosis (Figure 2). Protein and lipid trafficking via ER is universal (Cybulsky, 2017; Obacz et al., 2017), and vesicle-mediated PIP trafficking has been elucidated in roots of Arabidopsis following treatment with H_2_O_2_ (Wudick et al., 2015). The treatment induces AtPIP2;1 accumulation in the late endosomal compartments, and increases stability of the PIP and its homologs in the cytoplasm (Wudick et al., 2015). Like AtPIP1;4 (Li et al., 2015, 2019), AtPIP2;1 also is an H_2_O/H_2_O_2_/CO_2_ triple channel (Heckwolf et al., 2011; Rodrigues et al., 2017), but no study shows whether or not AtPIP2;1 resembles AtPIP1;4 to regulate bacterial effector translocation. It is deserved of studying whether multiple substrate specificities of a PIP enable it to accommodate bacterial effectors.

There are two examples indicating the possibility that T3 effectors of plant-pathogenic bacteria are translocated along with membrane trafficking. One is the T3 effector HopZ1a of *Pseudomonas syringae* pv. *syringae*, bacterial pathogen of many plants. HopZ1a, HopZ1b, and HopZ1c are allelic forms, constitute the HopZ1 family of *P. syringae* T3 secretion system, and share a consensus myristoylation site required for membrane localization (Zhou et al., 2009). HopZ1a is an acetyltransferase, is activated by the eukaryotic co-factor phytic acid, and turns to acetylate itself and tubulin. Tubulin acetylation causes a decrease in microtubule networks, disrupts the secretory pathway, and suppresses cell wall-associated defense in plants (Lee et al., 2012). The defense is subject to complex regulatory networks, which involve vesicle trafficking linked to microtubules (Lehman et al., 2017). The other example is the T3 effector HopM1 of *P. syringae* pv. *tomato*. To infect tomato plants, the bacteria secrets HopM1, and delivers it into the plant PM-derived trans-Golgi network/early endosome (Nomura et al., 2011), suggesting a role of vesicle trafficking in HopM1 translocation.

The involvement of AQPs in T3 effector endocytosis can be speculated from independent studies summarized below. The trafficking of animal AQPs towards the cell interior is triggered by the AQP binding to a different protein (Zelazny et al., 2009; Ji and Dong, 2015a), such as vasopressin (Kamsteeg et al., 2006), or heat shock protein HSP70 (Lu et al., 2007). Nevertheless, molecular interactions at the PM transiently affect PM integrity (Laliberté and Sanfaçon, 2010; Guignot and Tran Van Nhieu, 2016; Santi-Rocca and Blanchard, 2017), which may extricate and internalize PM-associated proteins to accommodate foreign molecules like T3 effectors. It is possible that AvrBs3 and PthXo1 use this mechanism to enter rice cells together with OsPIP1;3 trafficking (Figure 2). Both effectors may be internalized through trafficking of OsPIP1;3 en route to degradation by the proteasome (Hirano et al., 2003; Centrone et al., 2017) or the autophagosome (Khositseth et al., 2017). This mode of trafficking and degradation has been shown to regulate animal AQP turnover (Khositseth et al., 2017; Shen et al., 2019) and may also apply to plant AQPs. It is necessary to verify whether OsPIP1;3, or any other PIPs, can interact with any of the bacterial effectors, in the absence of Hpa1, to cause the PIP and effector internalization.

How could PM binding lead to endocytosis of bacterial effectors? The binding of effectors or translocators to the PM induces transient damage to the integrity and function of PM compositions, providing an abnormal pathway for bacterial effector translocation (Guignot and Tran Van Nhieu, 2016). In addition to T3, other secretion systems, such as T4, may be involved also (Domingues et al., 2016). *Salmonella enterica* serovars are intracellular facultative pathogens with a wide host range, and cause serious diseases including typhoid fever and cholera in humans (Domingues et al., 2016; Piscatelli et al., 2016). About 40 different T3 effectors confer differential virulence to different serovars. For infection, *Salmonella* bacteria establish a bacteria-containing vacuole (BVC), induce tubules, and then deliver the T3 effector SteA onto the BCV and tubules. In both structures, SteA specifically interacts with PI4P to move into host cells (Domingues et al., 2016). *Legionella pneumophila*, the pathogen responsible for Legionnaire’ disease, creates BCV through effectors secreted by the Dot/Icm T4 system (Finsel and Hilbi, 2015). In BCV, the pathogen hijacks host PM trafficking to induce BCV maturation (Hubber and Roy, 2010). The BCV membrane mainly contains PI4P (Weber et al., 2006; Finsel and Hilbi, 2015), which is important for anchoring many Dot/Icm effectors onto BCV (Dong et al., 2016). The T4 effector LepBd of *L. pneumophila* is a phosphatase (PP), and specifically converts PI3P into PI(3,4)P_2_. PI(3,4)P_2_ is efficiently hydrolyzed into PI4P (Dong et al., 2016), which may be used to replenish the PI4P stock of BCV. This mechanism is also employed by the T3 effector SopB of cholera pathogen *S. enterica* serovar Typhimurium. Like the T4 effector LepBd of *L. pneumophila*, the T3 effector SopB of Typhimurium is also a PP, but possesses both 4-PP and 5-PP activities. This dual enzymatic function is essential for the formation of BCV membrane ruffles and subsequent bacterial invasion. The 5-PP activity of SopB is assumed to generate PI(3,4)P_2_, which is then recruited by sorting nexin 9 (SNX9), an actin-modulating protein. The 4-PP activity converts PI(3,4)P_2_ to PI3P. Alone, neither activity is sufficient for membrane ruffling. Instead, combined 4-PP and 5-PP activities induce SNX9-mediated membrane ruffling and bacterial invasion (Dong et al., 2016).

The three models of T3ET may be chosen to use circumstantially by bacteria with genetic variations in the T3 repertoire. For example, the translocon-dependent mechanism guarantees efficient translocation of AvrBs3 from the wild-type *Xcv* strain (Büttner et al., 2002), in contrast to insufficient translocation from the bacterial translocon mutants in a translocon-independent manner (Scheibner et al., 2017). An early report stated that the carboxy (C)-terminal region of HrpF is essential for the entry of *Xcv* AvrBs3 into plant cells, whereas the nitrogen (N)-terminal contains a secretion signal and has no effect on effector translocation (Büttner et al., 2002). This suggests that xanthomonads T3ET occurs in a translocon-dependent manner. By contrast, a recent report proposed a translocon-independent pathway (Scheibner et al., 2017). The N-terminal 10 and 50 amino acids are required for T3 secretion and AvrBs3 translocation, respectively. Additional signals in the N-terminal 30 amino acids and the region between amino acids 64 and 152 promote AvrBs3 translocation. AvrBs3 translocation occurs in the absence of the T3 secretion chaperon HpaB, and in the absence of HrpF, which is a predicted component of the T3 translocon assembly. The authors suggested that the delivery of AvrBs3 begins during the early stages of infection, before the activation of HpaB or translocon integration into the plant PM (Scheibner et al., 2017). It is more likely that a different translocator, present in reserve and lacking function when the bacteria possesses a workable HrpF, is employed when HrpF loses function or is removed from the bacterial proteome.

## A Cytological Gap Between H_2_O_2_ Signaling and Immunity Pathways

H_2_O_2_ is stable compared with other ROS molecules such as the superoxide anion O_2_^–^ and hydroxyl radical OH^–^. In plants, H_2_O_2_ is produced by the enzymatic activities via multiple biochemical mechanisms (Smirnoff and Arnaud, 2019). These mechanisms include electron leakage from the electron transport chain in chloroplasts and mitochondria, the activity of peroxisomal oxidases and peroxidases in cytoplasm or plant cell walls, as well as the activity of NADPH oxidases (NOXs) in the PM (Smirnoff and Arnaud, 2019). The rapid production of ROS, especially H_2_O_2_, indicates the successful recognition of pathogen infection and molecular patterns (Alvarez et al., 1998; Torres, 2009). Well-known examples of pathogenic patterns include invariant microbial epitopes, such as fungal chitin (Kaku et al., 2006) and bacterial flagellin (Zipfel et al., 2004) and harpin proteins (Sang et al., 2012; Choi et al., 2013). These pattern molecules can be recognized by pattern receptors within the PM, which induce immune responses, including H_2_O_2_ production, in plants (Levine et al., 1994; Ausubel, 2005; Galletti et al., 2011).

The production of H_2_O_2_ is typically apoplastic, resulting mainly from the enzymatic activity of NOXs located in PMs (Sagi and Fluhr, 2006; Kärkönen and Kuchitsu, 2015; Smirnoff and Arnaud, 2019). Then, there is crosstalk between H_2_O_2_ and immunity pathways, such as systemic acquired resistance (SAR) and pattern-triggered immunity (PTI) to regulate plant disease resistance (Torres, 2009). SAR is characteristic of the induced expression of pathogenesis-related (PR) genes, typically *PR-1* and *PR-2*, under the regulation of non-inducer of *PR* genes-1 (NPR1) (Cao et al., 1997; Kim et al., 2011). NPR1 functions through conformational changes under cytoplasmic redox conditions (Tada et al., 2008) and through proteasome-mediated turnover in the nucleus (Spoel et al., 2009). The PTI pathway activates a cytoplasmic MAPK cascade (Asai et al., 2002), including a branch in which MPK3 and MPK6 phosphorylate different substrates (Bigeard et al., 2015; Pitzschke, 2015) to activate immune responses, including H_2_O_2_ and callose production (Bethke et al., 2012; Daudi et al., 2012). Callose is a β-1,3-glucan synthesized by glucan synthase-like (GSL) enzymes, with GSL5 playing a critical role in cellular immune responses (Lü et al., 2011). Therefore, both the SAR and PTI pathways comprise pivotal tiers of intracellular responses in the crosstalk with H_2_O_2_ produced in the apoplast (Sagi and Fluhr, 2006). It is clear that a cytological gap exists between H_2_O_2_ generation and functional performance. In fact, it remains unclear for a long time how apoplastic H_2_O_2_ penetrates plant PMs to enter the cytoplasm and regulate immunity.

## PIP-Mediated H_2_O_2_ Transport and Its Immunological Importance

**FIGURE 3:**
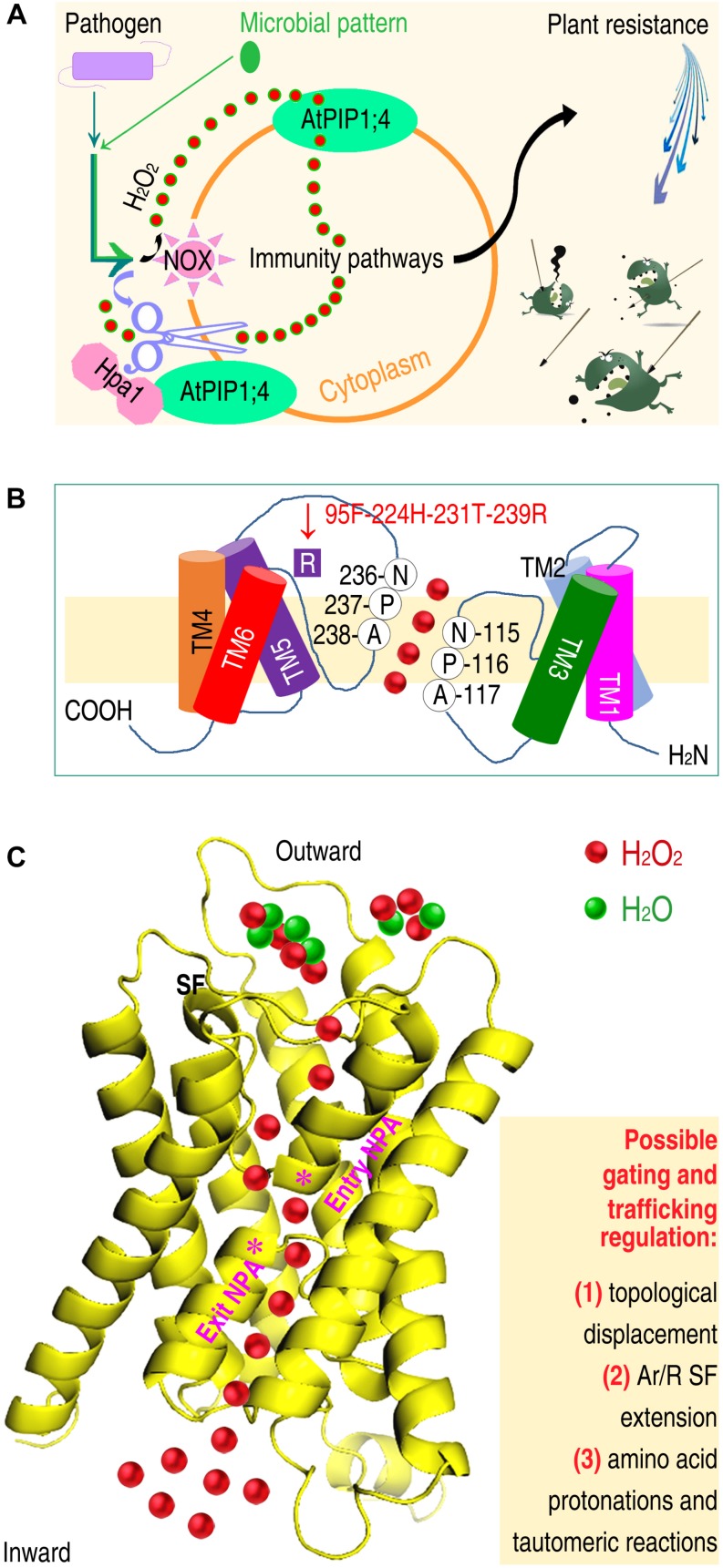
Crosstalk of AtPIP1;4-mediated H_2_O_2_ transport with the intracellular immunity pathways and predicted mechanisms by which AtPIP1;4 fulfills the substrate transport function. **(A)** Plant sensing of a pathogen or microbial pattern not only is an essential step of apoplastic generation and cytoplasmic import of H_2_O_2_, but also induces damages to the PM integrity (Guignot and Tran Van Nhieu, 2016). Impairment of the PM integrity is likely to provide an abnormal channel, which is wider than the normal conduit, and capable of accommodating substrates larger than H_2_O. **(B)** Hypothetic determinants of AtPIP1;4 conformation for H_2_O_2_ transport include amino acid compositions and locations in the NPA and SF regions. **(C)** Gating and trafficking regulation of the AtPIP1;4 channel for H_2_O_2_ transport across plant PMs (left) may be subject to the annotated factors (right). The 3D-structure of AtPIP1;4 was predicted by using the PHYRE2 (Protein Homology/analogy Recognition Engine V 2.0) program (http://www.sbg.bio.ic.ac.uk/phyre2/html/page.cgi?id~=~index). The diagrammatic transport of H_2_O_2_ over H_2_O is a surmise, predicted to occur by the combined mechanisms indicated on right.

Hpa1, applied to plants or produced in transgenic plants, functions as a bacterial pattern to activate the PTI and SAR pathways (Tian et al., 2016). Both pathways are activated by the generation of ROS, especially H_2_O_2_, in plant apoplasts. In Arabidopsis, inoculation with the bacterial pathogen *Pseudomonas syringae* pv. *tomato* or treatment with bacterial patterns, including Hpa1 and the flagellin functional fragment flg22, induce H_2_O_2_ generation in the apoplast. This H_2_O_2_ moves quickly into the cytoplasm, where H_2_O_2_ associates with PTI and SAR signal transduction. AtPIP1;4 serves as a H_2_O_2_ transport channel to facilitate apoplastic H_2_O_2_ import into the cytoplasm (Figure 3A), bridging the cytological gap in immunity signaling cascades (Tian et al., 2016).

This finding validates the hypothesis that H_2_O_2_ transport across a biomembrane is mediated by particular AQP isoforms in addition to certain membrane lipids (Bienert et al., 2006, 2007; Bienert and Chaumont, 2014; Aguayo et al., 2015). AQPs are transmembrane channels essential for the transport of H_2_O, H_2_O_2_, and other small substrates in all living cells (Maurel, 2007; Gomes et al., 2009). In this way, AQPs can modulate many physiological and/or pathological processes (Maurel, 2007; Ji and Dong, 2015a, 2015b; Li et al., 2019; Pawłowicz and Masajada, 2019; Zhang et al., 2019). In most plant species, five major families of AQPs exist. The PIP family has 11 members, PIP1;1–5 and PIP2;1–8 (Gomes et al., 2009; Abascal et al., 2014; Maurel et al., 2015). These are believed to mediate the transport of different substrates across plant PMs in an overlapping or redundant substrate-specific manner (Maurel, 2007; Péret et al., 2012, 2013; Prado et al., 2013). To date, five AtPIP isoforms (2;1, 2;2, 2;4, 2;5, and 2;7) are assumed to mediate H_2_O_2_ transport in engineered yeast cells (Bienert and Chaumont, 2014). The *de novo* expression of these PIPs can increase H_2_O_2_ sensitivity and decrease the viability of yeast (Dynowski et al., 2008; Hooijmaijers et al., 2012). Based on incomplete literature search, not all PIPs whose expression increases H_2_O_2_ sensitivity and decreases the viability of yeast have been verified for the H_2_O_2_ transporting function. AtPIP2;1 was determined to increase H_2_O_2_ uptake by yeast cells (Dynowski et al., 2008; Bienert and Chaumont, 2014) and by Arabidopsis guard cells (Rodrigues et al., 2017). AtPIP1;4 has been shown to function in H_2_O_2_ transport from the apoplast into the cytoplasm of Arabidopsis (Tian et al., 2016). Many works are required to test *in planta* function of the AQP candidates in H_2_O_2_ translocation.

## Conservative AQP Function for H_2_O_2_ Transport

AtPIP1;4 was determined to have triple substrate specificities (Li et al., 2015; Tian et al., 2016). In addition to transporting H_2_O_2_ (Tian et al., 2016), AtPIP1;4 partakes in the cellular hydraulic conductance (P_f_) of roots, and in mesophyll conductance of CO_2_ (*g*_m_); however, it does not affect stomatal CO_2_ conductance (*g*_s_) or P_f_ in leaves (Li et al., 2015). The interaction of AtPIP1;4 with Hpa1 at Arabidopsis PMs promotes substrate transport, increasing the net photosynthesis rate (*A*_N_), while P_f_ is also increased in leaves and roots (Li et al., 2015). Therefore, a PIP can alter its physiological functions or effect extents in response to plant pathogens or bacterial patterns.

The function of AtPIP1;4 in immunity is an extension of its primary roles in substrate transport, which was initially assigned to AQPs in mammals (Preston and Agre, 1991) and subsequently in plants (Maurel et al., 1993). The functional extension of AtPIP1;4 has biological importance for at least two reasons. First, AtPIP1;4-dependent SAR responses induced by bacterial pathogens effectively repress pathogen virulence (Tian et al., 2016; Figure 3A). In this case, pathogen-associated repressors of plant immunity (Oh and Collmer, 2005; Zhang et al., 2007; Guo et al., 2009) may be inhibited, or their immunity-repressing functions may be counteracted by the role of AtPIP1;4 in H_2_O_2_ translocation, which is linked to the immunity pathway. Second, AtPIP1;4 is an integral component of PTI in response to typical patterns, with conserved microbial cell-surface composition, i.e., flagellin (Zipfel et al., 2004) and chitin (Kaku et al., 2006). Despite their different biochemical nature, both patterns require AtPIP1;4 to induce PTI responses, except in the absence of induced *MPK6* expression (Tian et al., 2016). This is consistent with previous findings that the MAPK cascade diverges at MPK3 and MPK6 (Asai et al., 2002; Bigeard et al., 2015) to regulate distinct substrates in response to different patterns (Galletti et al., 2011; Pitzschke, 2015). Moreover, the induction of *MPK3* expression represents a circuit of the MAPK cascade in response to H_2_O_2_ (Gudesblat et al., 2007). These sets of information suggest that AtPIP1;4 plays a prominent role in immunity signaling by mediating apoplastic H_2_O_2_ translocation into plant cells.

AQP-mediated H_2_O_2_ transport in immune signaling also occurs in mammals. Among 13 AQPs, AQP3 is a H_2_O_2_ transport channel (Miller et al., 2010). AQP3-mediated H_2_O_2_ transport is associated with necrosis factor-κB (NF-κB) signaling in keratinocytes, and in the pathogenesis of psoriasis in response to cytokine regulation (Hara-Chikuma et al., 2015). The induction of psoriasis by cytokines, NF-κB activation, and intracellular H_2_O_2_ accumulation are concomitantly reduced in AQP3-knockout mice. In primary keratinocyte cultures, H_2_O_2_ is generated by membrane-associated NOX2 in response to TNF-α, and moves into intracellular spaces. Cellular import of H_2_O_2_ is facilitated by AQP3 and is required for NF-κB activation under PP2A regulation. Since AQP3 associates with NOX2 at PMs, this interplay may constitute H_2_O_2_-mediated signaling in response to TNF-α stimulation (Hara-Chikuma et al., 2015). Moreover, under oxidative stress, AQP3-mediated H_2_O_2_ transport attenuates apoptosis by regulating the P38 MAPK pathway (Xu et al., 2018; He and Yang, 2019). Based on these findings, and those regarding PIPs, cytoplasmic import across the PM can reduce the cytological distance for H_2_O_2_ generation, and functional performance (Bienert et al., 2006; Sang et al., 2012; Tian et al., 2016). Apocytoplastic signaling is conserved in plants and animals.

## AQP Structure for H_2_O Transport

It is unclear how different AQPs function in the transport of corresponding substrates, and how an AQP, such as AtPIP1;4 (Li et al., 2015; Tian et al., 2016), can function as a triple substrate transport conduit. One hypothesis is that structural details allow for differences in selectivity and modes of regulation (Kreida and Törnroth-Horsefield, 2015). Regarding H_2_O_2_ transport, the structures of AQP/PIP channels have not been studied, but can be inferred from information on structures of AQPs that function as water channels.

Plant aquaporins are predominant channels of H_2_O transport between the outside and inside of the cell, and between intracellular organelles (Huang et al., 2017). Although cotransporters and uniporters have been implicated in water homeostasis, AQPs have been accepted as intramolecular channels for the transmembrane movement of H_2_O down an osmotic gradient (Maurel et al., 2015; Huang et al., 2017; Yang, 2017; Pawłowicz et al., 2018). H_2_O transport by AQPs is determined by their three-dimensional structure.

Structural studies have characterized AQPs as homotetramers, which are integrated into the membrane with conserved configurations (Fu et al., 2000; Sui et al., 2001; Törnroth-Horsefield et al., 2006; Horsefield et al., 2008; Eriksson et al., 2013; Kirscht et al., 2016). Each monomer has a functional pore formed by six α-helical TM domains (TM1–TM6), five connecting loops (LA–LE), and two shorter helices (HB and HE). The outward end of HB and inward end of LE contain a pair of asparagine (N), proline (P), and alamine (A) tandem (NPA) motifs, which constitute the central channel through the membrane (Kirscht et al., 2016). Two NPAs form a conical funnel or traditional hourglass, which are linked at the tip and open outward from LE and inward from TM5 (Törnroth-Horsefield et al., 2010), and are essential for AQP function (Wree et al., 2011; Chen et al., 2018). Within LE, TM2 and TM5, the aromatic/arginine (Ar/R) selective filter (SF) is formed by four residues including aromatic amino acids and an arginine (R) residue; hence its name (de Groot et al., 2003). The SF is located in the outward opening of the channel and allows H_2_O to pass while repelling protons and cations (Eriksson et al., 2013). Multiple physical factors, such as hydrophilic and hydrophobic interactions, electrostatic repulsion, and dipole alignment between amino acid residues within or around the NPA and SF, influence substrate selectivity (Törnroth-Horsefield et al., 2006).

A pivotal step toward the substrate-transporting function of AQPs is the regulation of gating (opening and closing) and trafficking (substrate transport). This has been elucidated for water channels at angstrom (Å) or sub-Å resolution (Daniels et al., 1999; Fotiadis et al., 2001; Kukulski et al., 2005; Kreida and Törnroth-Horsefield, 2015). Considering spinach *Spinacia oleracea* SoPIP2;1, channel opening is triggered by the phosphorylation of conserved serine (S) 197 (Johansson et al., 1996; Kukulski et al., 2005), and is expedited by hydrogen bond networks in LD (Törnroth-Horsefield et al., 2010). Channel closure results from the dephosphorylation of S115 in LB and S274 in the C-terminal region of the AQP sequence under conditions of drought stress, or from the protonation of a conserved histidine (H) residue following a decrease in cytoplasmic pH due to anoxia during flooding. Dissection of SoPIP2;1 crystal structures, both the closed conformation at 2.1 Å and the open conformation at 3.9 Å, reveals the importance of LD displacement for gating and trafficking. The dephosphorylation of S115 and S274 prevents outward NPA entry from LB, and inward NPA exit in TM5. In the open conformation of SoPIP2;1, S197 is phosphorylated at LD, LD is displaced up to 16 Å, the nitrogen terminus of TM5 extends a further half-turn into the cytoplasm, and NPA entry and exit are promoted. In addition, H193 protonation and interactions between amino acids, including hydrogen bond networks and electrostatic repulsion, also influence the switch between opening and closing of the channel (Törnroth-Horsefield et al., 2010).

Crystal structure analysis of Aqy1, the only AQP in yeast *Pichia pastoris*, at a sub-Å (0.88 Å) resolution, provides evidence for tautomeric reactions to expedite H_2_O transport (Eriksson et al., 2013). Hydrophilic amino acids in NPA and SF interact to bind H_2_O molecules, which are then navigated through the channel. With polar hydrogen bond configurations, four H_2_O molecules per group pass the SF, and then divide into two pairs to pass through the inward NPA region. There are two types of tautomerism between hydrophilic amino acids in the SF. One is proton transfer – the atom Nδ, but not Nε, of H212 is protonated to provide a proton for L208, with the role of guiding H_2_O movement. The other one is covalent binding – atoms Cζ and Nη2 of R227 maximally bind to each other, Nη2 is closest to the central conduit, and its positive charge repels cations, creating favorable conditions for H_2_O to travel through the SF. With this advantage, four compact H_2_O molecules are located within the full space of the SF, where they synchronize to move within and across the SF passage. Due to high impacts of atom tautomerism and hydrogen-bond interactions restricted to the H_2_O molecules in transport, other H_2_O molecules must wait for the next round of the channel opening and trafficking, and proton or cations are unable to enter the SF.

In addition to the structural configuration, biochemical regulation is also indispensable to the function of AQPs. In this aspect, channel gating and trafficking regulation by phosphorylation are ubiquitous for all AQPs (Li and Wang, 2017; Kapilan et al., 2018; Laloux et al., 2018; Nesverova and Törnroth-Horsefield, 2019). Additionally critical mechanisms underlying the functional regulation of different AQPs include biotic and abiotic signals. They induce the transport of different substrates (Tian et al., 2016; Ruiz-Lozano and Aroca, 2017; Balestrini et al., 2018; Smirnoff and Arnaud, 2019) by stimulating AQPs themselves with gradients over membranes and by interacting with other proteins (Ji and Dong, 2015a; Roche and Törnroth-Horsefield, 2017). These have been topics of many literatures (for example: Maurel and Plassard, 2011; Hara-Chikuma et al., 2015; Ji and Dong, 2015a; Maurel et al., 2015; Yang, 2017; Roche and Törnroth-Horsefield, 2017) and will not been discussed in this article.

## Control of Substrate Specificities

This is a question for AQPs capable of transporting substrates other than H_2_O, especially those that have multiple permeation properties. In addition to H_2_O, approximately 20 other substrates require AQPs to move between the exterior and interior of cells, and between organelles (Laloux et al., 2018). A fifth pore created by four AQP monomers of a homotetramer in the lipid bilayer (Wang et al., 2007) or yeast membrane (Otto et al., 2010) has been proposed for gas (CO_2_ and O_2_) and ion transport (Kaldenhoff et al., 2014). Moreover, many AQPs have more than one substrate (Kreida and Törnroth-Horsefield, 2015; Maurel et al., 2015; Fox et al., 2017; Laloux et al., 2018). Examples include AtPIP2;1 for H_2_O/H_2_O_2_ (Dynowski et al., 2008; Verdoucq et al., 2008), AtPIP1;4 for H_2_O/H_2_O_2_/CO_2_ (Li et al., 2015; Tian et al., 2016), and TIPs for H_2_O, H_2_O_2_ and/or ammonia (NH${}_{3}$; Maurel et al., 1993; Loque et al., 2005; Bárzana et al., 2014) transport. Regulation of gating and trafficking must differ considerably between specialist channels, different generalist channels, and channels for H_2_O and a different substrate. Variation in NPA diameter, the composition and width of SF, neighboring residues, and their interactions with each other and with the substrate might explain multiple functions of AQPs/PIPs in the transport of different substrates, and the multiple substrate transport capacities of a single AQP/PIP (Fox et al., 2017).

Recently, a smart solution was proposed in a study on the 1.18 Å crystal structure of AtTIP2;1 (Kirscht et al., 2016). That study characterized AtTIP2;1 as an NH_3_ transport channel, which functions with an extended SF. The channel diameter in the NPA region is smaller than that of other AQPs, but remains constant at ∼3Å along the channel; this is in contrast to the narrowing of SF in other AQPs. The topological positions of four SF residues in TM2, TM5, LE, and HE are thought to determine substrate selectivity (de Groot et al., 2001). Consistent with this model, TIP2s deviate from other AQPs in terms of the wider SF, which is mainly caused by an isoleucine (I185) in TM5, replacing a histidine that is conserved in water-specific AQPs (Kirscht et al., 2016). The most striking feature of the SF in AtTIP2;1 is the R200 located in HE, while the arginine in HE is conserved in most AQPs. In AtTIP2;1, the R200 side chain is located at the edge of the channel due to the H131 situated in LC, making histidine the fifth residue of the extended SF. The position of this arginine is further stabilized by a hydrogen bond with histidine (H63) in TM2, which occupies the same space as the corresponding aromatic residues of water and glycerol channels without direct effects on the channel opening (Kirscht et al., 2016). Moreover, H131 in LC interacts directly with the substrate in the selectivity region. These structural features define the extended SF at five positions: I185, R200, H131, and H63, which have properties and configurations that establish the novel SF, plus G191 in LE, which is conserved in the canonical and extended SF. The concept of extended SF is instructive to conceiving study schemes before initiating analysis of APQ/PIP channels for transport of H_2_O_2_ and more substrates other than H_2_O and NH_3_.

## Structural Basis of PIPs for Mediation of H_2_O_2_ Transport

Until the structural basis of PIP/AQP functions in H_2_O_2_ transport is dissected, no more than inspiration can be deduced from referencing the crystal structures of SoPIP2;1 for NH_3_ transport (Kirscht et al., 2016) and both Aqy1 (Eriksson et al., 2013) and AtTIP2;1 (Törnroth-Horsefield et al., 2010) for H_2_O transport. The topological displacement of the connecting loop (Törnroth-Horsefield et al., 2010) may have a broad importance for AQPs. Tautomeric reactions (Eriksson et al., 2013) and the SF extension (Kirscht et al., 2016) might be used by certain PIPs/AQPs to expedite H_2_O_2_ transport. However, these features are likely to be insufficient to support H_2_O_2_ transport, due to the difference in diameter/molecular mass of H_2_O_2_ (3.70 Å/34), H_2_O (2.96 Å/33) and NH_3_ (<2.96 Å/17), and in the Ar/R SF features. The location and composition of the SF is identical (F87, H126, T225, R231) in the H_2_O_2_ channel AtPIP2;1 (Rodrigues et al., 2017) and the water channel SoPIP2;1 (Kirscht et al., 2016). However, the SF composition shared by AtPIP2;1 and SoPIP2;1 is distinct from that in the corresponding positions (G87, I126, L225, and T231) of the H_2_O_2_ channel AtPIP1;4 (Tian et al., 2016). AtPIP1;4 is the same length as OsPIP2;1, but possesses six more residues than SoPIP2;1, with a predicted Ar/R SF comprising F95, H224, T231, and R239 (Figure 3B). If the SF extension permits AQPs to mediate H_2_O_2_ transport, the degree of the SF extension must be considerably higher than that in the NH_3_ transport channel (Kirscht et al., 2016).

Three issues are considered to infer the structural basis of the function of PIPs in H_2_O_2_ transport between the outside and inside of plant cells. First, the apocytoplastic transport of H_2_O_2_ is more intricate as compared to the signal shift ways by the cell-to-cell traveling via plasmodesmata (Wang et al., 2009) and via vesicle-aided trafficking between organelles through the ER system within the cell interior (Ashtamker et al., 2007; Melo et al., 2017). Second, H_2_O_2_ transport in and out of plant cells is not constant throughout the life circle of plants (Dynowski et al., 2008; Tian et al., 2016). Third, H_2_O_2_ trafficking across the PM is induced but is not constitutive, and occurs only when apoplastic H_2_O_2_ is generated in response to pathogens, microbial patterns, or environmental signals (Levine et al., 1994; Xin et al., 2015; Liu and He, 2016; Tian et al., 2016).

Plasma membrane sensing of these distinct signals will promote H_2_O_2_ generation in apoplasts and its immediate translocation into the cytoplasm (Ausubel, 2005; Ashtamker et al., 2007; Tian et al., 2016) by three possible mechanisms. One is inductivity (Figure 3A). When plants are infected by a pathogen or respond to a microbial pattern, such as Hpa1 or flg22, the enzymatic activity of NOX is induced to catalyze the generation of H_2_O_2_ by peroxidation and superoxidation in PMs (Tian et al., 2016; Smirnoff and Arnaud, 2019). The generated H_2_O_2_ accumulates, and the concentration increases temporarily in the apoplast. This creates a gradient from the outside to the inside of the cell (Tian et al., 2016), and induces the PIP channel to function (Tian et al., 2016).

The second mechanism is speculated to be the combination of factors (Figure 3C) found in SoPIP2;1 (Törnroth-Horsefield et al., 2010), Aqy1 (Eriksson et al., 2013), and AtTIP2;1 (Kirscht et al., 2016). Combined factors facilitate the passage of H_2_O_2_ through the PIP channel, which could be established by SF extension (Kirscht et al., 2016), and optimized by amino acid protonation (Eriksson et al., 2013). H_2_O_2_ generation (2O_2_^–^ + 2H^+^ = H_2_O_2_) requires protons, and may reduce the likelihood that amino acid residues near the SF and NPA regions are protonated. As the protonation navigates H_2_O movement along the channel (Eriksson et al., 2013), decreased protonation will disturb H_2_O transport. This might promotes the transport of H_2_O_2_ over H_2_O through a PIP channel once a sufficient diameter is reached (>3.70 Å).

The third mechanism is supposed to be biochemical responses (Figure 3C) associated with the regulation of PM remodeling – injury and repair (Laliberté and Sanfaçon, 2010; Santi-Rocca and Blanchard, 2017). PM remodeling is triggered by the binding of an active extrinsic protein, including microbial patterns such as Hpa1 (Li et al., 2015; Tian et al., 2016), bacterial T3 translocators such as HrpF (Büttner et al., 2002; Li et al., 2011), and bacterial effectors (Hubber and Roy, 2010; Domingues et al., 2016; Dong et al., 2016). Binding of these bioactive proteins affects the PM integrity (Ji and Dong, 2015a; Guignot and Tran Van Nhieu, 2016). Reduced PM integrity is advantageous for solute influx, which, however, is strictly regulated by proteins and lipids that recognize microbial patterns, T3 effectors, or translocators (Gilbert et al., 2014).

The former two mechanisms may synergize in the gating and trafficking regulation, requiring AtPIP1;4 to transport H_2_O_2_ in plants grown under regular conditions without any input signal, except for externally applied H_2_O_2_ or H_2_O_2_ induced by a pathogen or a microbial pattern (Tian et al., 2016). The third mechanism may occur in the presence of Hpa1 following application to plants or production in transgenic plants, in which AtPIP1;4 interacts with Hpa1 (Li et al., 2015) to increase the substrate transport function. Studies should aim to verify this hypothesis in order to elucidate the structures of PIP orthologs as transport channels for H_2_O_2_ or different substrates.

## Conclusion and Perspectives

Finite research performed on these case studies is based on a solid foundation obtained through extensive studies; research on the structural regulation of PIP function in plant infection and immunity is invited. The first case study on Hpa1-mediated, OsPIP1;3-associated, and virulence-relevant PthXo1 translocation offers multiple experimental avenues to characterize interactions between T3 translocators and their receptors at target PMs, as well as the associated implications for effector translocation and virulence. The two subjects discussed here are yet to be thoroughly studied. First, which of the assumed delivery lanes is used by different effectors is a long-standing question for all plant-pathogenic bacteria. Xoo possesses more than 30 effectors secreted by the T3 system (White et al., 2009), similar to the number in other bacteria. Further study is needed to identify all T3 effectors in the three proposed mechanisms: translocon-independent pore formation (Figure 1), endocytosis with PM protein or lipid trafficking (Figures 1, 2), and translocon-dependent delivery (Figure 1). The second subject includes the contribution of PM lipids and proteins to T3 effector translocation. T3 translocon assembly or pore formation must recruit both lipids and proteins situated in plant PMs (Büttner et al., 2008; Gilbert et al., 2014; Heilmann and Heilmann, 2015; Ji and Dong, 2015b; Guignot and Tran Van Nhieu, 2016). It would be of great interest to determine how effectors are internalized with PM protein or lipid trafficking, and how protein and lipid receptors of T3 translocators coordinate their actions to generate pores or translocons in plant PMs.

The second case study discusses AtPIP1;4-regulated, Hpa1-promoted, and immunity-linked H_2_O_2_ transport, and establishes a cytological connection between the generation and function of H_2_O_2_ in the apoplast and cytoplasm, respectively (Tian et al., 2016). The cytoplasmic import of H_2_O_2_ bridges a physical gap, which was unknown for at least 20 years since the biphasic H_2_O_2_ accumulation following induction was awarded biological significance (Levine et al., 1994). AtPIP1;4-mediated H_2_O_2_ translocation is a pivotal step in apocytoplastic signal transduction for intracellular immunity pathways, which regulate SAR and PTI responses, leading to plant resistance against diseases (Dong et al., 1999; Chen et al., 2008a, b; Choi et al., 2013; Zhao et al., 2014). The future focus of studies will be difficult, highlighting the regulation of gating and trafficking of the AtPIP1;4 channel for H_2_O_2_ transport. To date, the structures of AQP channels have only been determined for the transport of NH${}_{3}$ (Kirscht et al., 2016) and H_2_O (Daniels et al., 1999; Fotiadis et al., 2001; Kukulski et al., 2005; Kreida and Törnroth-Horsefield, 2015), and almost 20 substrates remain to be understood (Laloux et al., 2018). Rational hypotheses on structural themes in both gating and trafficking (Kreida and Törnroth-Horsefield, 2015) requires the efforts of researchers to explore structural mechanisms that govern diverse AQP channels. It is necessary to dissect the conformation of AtPIP1;4 (Figures 3B,C) involved in H_2_O_2_ transport in response pathogens or patterns (Figure 3A). It is especially necessary to study whether the H_2_O_2_ transport is facilitated by combined impetuses, including the SF extension, amino acid residue interactions (Figure 3C), and PM protein trafficking (Figure 2).

The two case studies have been designed to converge at the intersection Hpa1-PIP cooperation and branch into two directions. One targets plant immunity, for which Hpa1 functions as a bacterial pattern in a pathogen-independent manner. The other contributes to plant infection, in which Hpa1 acts as a T3 translocator after secretion by the bacteria, and mediates the translocation of virulent effectors that lead to disease. These findings provide insight into disease control either through induced immunity, or the prevention of bacteria from usurping the substrate transport gate. Practical application of both strategies to strengthen crop protection (Krinke et al., 2007; Chen et al., 2008b; Fu et al., 2014; Wang D. et al., 2014; Li et al., 2019) will integrate with crop involvement by using AQPs from plants themselves (Ruiz-Lozano and Aroca, 2017; Balestrini et al., 2018) and from symbiotic microbes as well (Kikuchi et al., 2016; Desaki et al., 2018).

## Author Contributions

LZ, LC, and HD drafted the manuscript. LZ predicted the 3D structure of AtPIP1;4. HD finalized the manuscript.

## Conflict of Interest Statement

The authors declare that the research was conducted in the absence of any commercial or financial relationships that could be construed as a potential conflict of interest.
